# Rotavirus-associated mild encephalopathy with a reversible splenial lesion (MERS)—case report and review of the literature

**DOI:** 10.1186/s12879-015-1192-5

**Published:** 2015-10-24

**Authors:** Konstantinos Karampatsas, Christina Spyridou, Ian R. Morrison, Cheuk Y. W. Tong, Andrew J. Prendergast

**Affiliations:** Department of Paediatrics, The Royal London Hospital, Barts Health NHS Trust, London, UK; Department of Virology, The Royal London Hospital, Barts Health NHS Trust, London, UK; Centre for Paediatrics, Blizard Institute, Queen Mary University of London, London, UK; Queen’s Hospital, Barking, Havering and Redbridge University Hospitals NHS Trust, Romford, UK

**Keywords:** Rotavirus, Encephalopathy, Central Nervous System Infection, Corpus Callosum, MERS, Paediatrics

## Abstract

**Background:**

Rotavirus is the most common cause of severe gastroenteritis in children under the age of 5 years worldwide. It is well recognised that rotavirus can cause signs and symptoms beyond the gastrointestinal tract, including neurological manifestations such as encephalopathy. Mild encephalopathy with a reversible splenial lesion (MERS) is a clinico-radiological syndrome that has been associated with rotavirus. We report a case of a 4-year-old boy with clinically mild encephalopathy, who had an isolated splenial lesion in the corpus callosum on neuroimaging, and rotavirus RNA detected in faeces. We use this case as an opportunity to review the literature on rotavirus-associated MERS.

**Case presentation:**

A previously healthy 4-year-old boy presented with a 2-day history of vomiting, diarrhoea, and fever, complicated by reduced level of consciousness. Magnetic resonance imaging of the brain showed a marked hyperintensity in the splenium of the corpus callosum on T2 and diffusion-weighted images. Rotavirus genome was detected by polymerase chain reaction in a stool specimen, but not in the cerebrospinal fluid. The genotype was identified as G1P8. His clinical condition improved with gradual resolution of his symptoms. No neurological complications were evident upon discharge and the patient had no recurring symptoms or significant residual defects when followed up 2 months later.

**Conclusion:**

MERS is a novel clinic-radiological syndrome first described in Japan. A transient splenial lesion with reduced diffusion that appears as a high signal intensity in diffusion-weighted MRI is the main diagnostic feature. Rotavirus is one of the most common agents associated with MERS, although to our knowledge only one previous case has been reported from Europe. The majority of patients appear to achieve full recovery following rotavirus-associated MERS, irrespective of treatment. This case, together with other published reports, supports the hypothesis that rotavirus-associated MERS is unlikely to be the result of direct viral invasion of the CNS. It has been suggested that MERS may be caused by intra-myelinic axonal oedema or local inflammatory cell infiltration; however, the pathogenesis remains incompletely understood.

## Background

Rotavirus is the most common cause of severe gastroenteritis in children under the age of 5 years worldwide [1]. It is well recognised that rotavirus has a viraemic phase [2] and can cause signs and symptoms beyond the gastrointestinal tract, including neurological manifestations such as encephalopathy [3]. We present a case of a 4-year-old boy with clinically mild encephalopathy, who had an isolated splenial lesion in the corpus callosum on neuroimaging, and rotavirus RNA detected in faeces. We use this case as an opportunity to review the literature on a clinical entity termed rotavirus-associated mild encephalopathy with a reversible spenial lesion (MERS), providing insights into the pathogenesis, management, and prevention of this emerging condition.

## Case presentation

A previously healthy 4-year-old boy of Indian origin was admitted to our hospital with a 2-day history of vomiting, diarrhoea, and fever, complicated by sudden disturbance of consciousness on the day of admission. He was fully vaccinated (excluding rotavirus vaccine, which was not a routine component of the UK schedule at the time), and had no recent history of foreign travel or animal contact. On admission, he had a temperature of 39.6 °C, heart rate of 155 beats/min, respiratory rate of 30 breaths/min, oxygen saturation of 99 % in room air, and blood pressure of 92/59 mm Hg. He appeared mildly dehydrated. He was drowsy with transient periods of agitation and had a Glasgow Coma Scale score of 13 (eyes 4, verbal 4, motor 5). His parents reported that he was awake but lethargic. He was unable to recognise them and lost the ability to make eye contact. He looked confused, uttered meaningless or incoherent words and phrases and could not respond to questions in an appropriate manner.

**Fig. 1:**
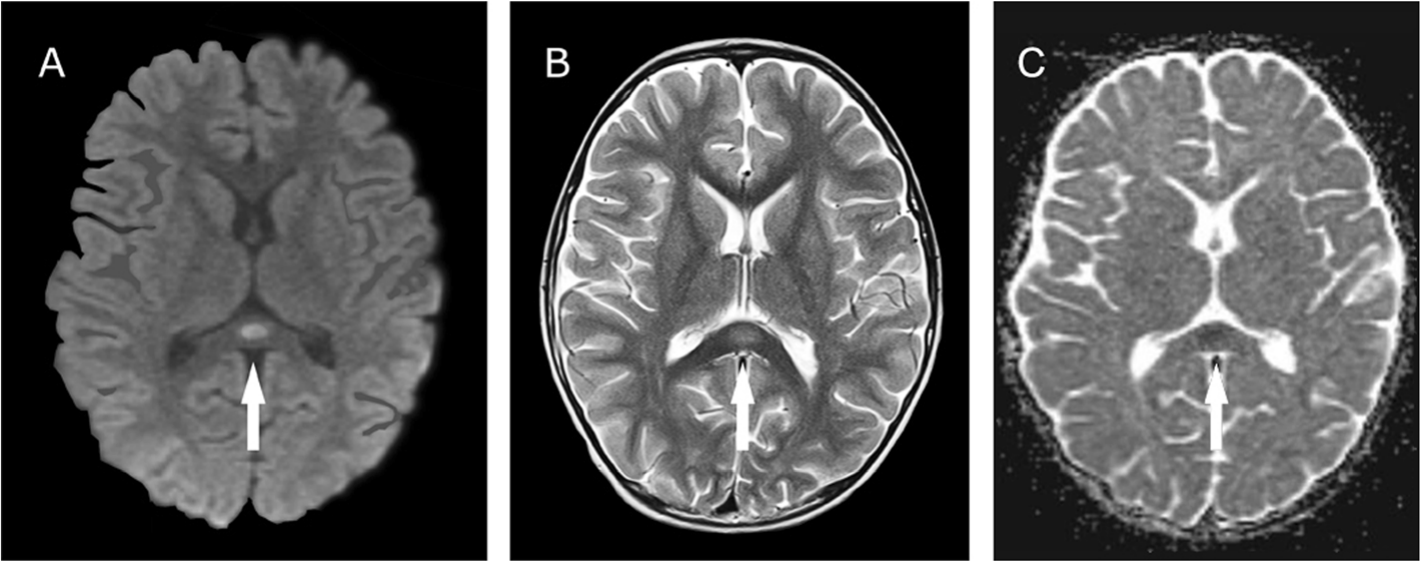
MRI of the brain performed on day 6 of disease **a** Diffusion-weighted image shows a marked hyperintensity in the SCC. **b** Corresponding T2 image. **c** Corresponding diffusion restriction on Apparent Diffusion Coefficient (ADC) mapping

Laboratory blood testing showed haemoglobin 11.9 g/dL, leucocyte count 11,100 cells/μL, platelet count 225,000 cells/μL, serum sodium 131 mmol/L, potassium 4.3 mmol/L, chloride 92 mmol/L, urea 8.3 mmol/L, creatinine 54 μmol/L, alanine aminotransferase 105 U/L, ammonia 85 μmol/L, and C-reactive protein 25 mg/L. Cerebrospinal fluid (CSF) examination revealed no leukocytes, protein 0.15 g/L, and glucose 3.6 mmol/L. Cranial computed tomography (CT) showed no evidence of an intracranial lesion. Magnetic resonance imaging (MRI) of the brain performed on day 4 of hospitalization (6 days after his symptoms began) showed a marked hyperintensity in the splenium of the corpus callosum (SCC) on T2 and diffusion-weighted images with corresponding diffusion restriction on Apparent Diffusion Coefficient (ADC) mapping (Fig. 1). Initial electroencephalograph (EEG) 2 days after admission exhibited widespread, excessive slow wave activity, more prominent in the right hemisphere. The patient did not experience any seizures, and the abnormality was not evident on a subsequent EEG performed 30 days after discharge. Rotavirus RNA was detected by polymerase chain reaction (PCR) in a stool specimen, but was not detected in CSF. The rotavirus genotype was identified as G1P8.

An infusion of intravenous isotonic fluid (0.9 % sodium chloride and 5 % glucose) was administered. Intravenous ceftriaxone, clarithromycin, and aciclovir were started on the day of admission due to persistent disturbance of the level of consciousness and suspected acute encephalitis. His clinical condition improved over the subsequent 5 days, with gradual resolution of his symptoms, and antimicrobial treatment was discontinued once CSF culture confirmed no bacterial growth and PCR was reported negative for herpes simplex virus. No neurological complications were evident upon discharge. The patient had not had any recurrence of symptoms when seen for follow-up 8 weeks later. A follow-up MRI was therefore not performed.

## Discussion

We describe a previously healthy 4-year-old boy presenting with rotavirus gastroenteritis complicated by encephalopathy. Although rotaviruses predominantly infect the small intestine, neurological manifestations are well described, including febrile convulsions, afebrile seizures, meningoencephalitis, cerebellitis, and encephalopathy [3]. In our case, the child’s illness was associated with a specific, isolated MRI lesion with restricted diffusion in the splenium of the corpus callosum. Tada and co-workers [4] first proposed a novel clinicoradiological syndrome in Japan in 2004, characterized by an encehalopathic illness associated with a high-signal intensity in the splenium. The clinical and radiological changes were found in association with a range of infections and were shown to be reversible, leading to the term Mild Encephalopathy/Encephalitis with Reversible Splenial Lesion (MERS), for which diagnostic criteria have been proposed (Table 1). In our case, the finding of acute onset of impaired consciousness following an episode of rotavirus gastroenteritis, in association with an isolated abnormal MRI signal in the splenium, reversible EEG changes, and rapid clinical recovery support a diagnosis of rotavirus-associated MERS, although we did not repeat an MRI scan in this child because his symptoms rapidly and completely resolved.

**Table 1:** Diagnostic criteria of MERS

1. Onset with neuropsychiatric symptoms, such as abnormal speech and/or behaviour, and impaired consciousness and convulsion, within 1 week after the onset of fever.
2. Complete recovery without sequelae, mostly within 10 days after the onset of neuropsychiatric symptoms.
3. High-signal-intensity lesion in the splenium of corpus callosum, in the acute stage. T1 and T2 signal changes are mild.
4. Lesion may involve the entire corpus callosum and the cerebral white matter in a symmetric fashion.
5. Lesion disappears within 1 week, with neither residual signal changes nor atrophy.

Source: (Hoshino et al. [6], 2012, p.338 Table 1)

We undertook a literature search to identify other reports (reviews, case reports or case series) of rotavirus-associated MERS, using the PubMed database with the search term “MERS”, “Encephalopathy”, “Encephalitis”, “Splenium of corpus callosum” and “Rotavirus”. Additional articles were extracted from the references lists of full publications. For the purpose of this review, we followed the diagnostic criteria for MERS cited in Table 1. We found 15 papers published between September 2002 and June 2015. One paper was excluded on the basis of language [5] and two papers were excluded because they provided no data on the clinico-radiological features of MERS [6, 7], leaving 12 reports that were included in this review (Fig. 2). We identified 10 case reports [8–17], one case series [18] and one comparative study [19] for a total of 13 cases of rotavirus-associated MERS in the English language [8–19]. The case reported by Kobata et al. [8] was published before the term MERS was introduced, but it was included in the review since it met the current criteria for MERS.

Of the 13 cases, 10 were from Japan, one from Korea, one from China and one from Poland. The median age at presentation was 2 years (range 1–6 years) with no sex difference (6 males: 7 females). In all cases, MERS was preceded by symptoms of gastroenteritis, such as vomiting, diarrhoea and fever. Hyponatraemia (Na <136 mmol/L) was reported in 6/13 (46 %) cases. Seizures (9/13; 69 %) and disturbance of consciousness (8/13; 62 %) were the most common neurological signs. Abnormal EEG changes were reported in 5/13 (38 %) cases, with diffuse slow waves in three and occipital slow waves in two. All abnormalities improved on repeat EEG testing.

Twelve patients (92 %) had an isolated lesion in the splenium of the corpus callosum, and one patient (8 %) had lesions in the splenium and genu of the corpus callosum. In all cases, the lesions completely reversed within 12 days (range 5–12 days). All patients recovered completely (Table 2). In 5 cases in which CSF was tested, rotavirus RNA PCR alone (*n* = 3) or in combination with antigen (*n* = 2) were negative; RNA and/or antigen were detected in the serum of two cases. Rotavirus was detected in stool samples from all children; in those for whom genotyping was performed, genotypes identified were G1 (*n* = 2), G3 (*n* = 2), G9 (*n* = 1), and G5 (*n* = 1).

**Table 2:** Rotavirus-associated MERS in children: review of the literature

Author, year, location, reference,	Sex, age (years)	Prodromal symptoms	Interval to initial neurological symptom (days)	CNS symptoms	Serum Na (mEq/l)	Stool detection of RV	Serum detection of RV	CSF detection of RV	Interval to disappearance of SCC lesion (days)	EEG findings	Treatment	Outcome
Kobata et al, 2002 Japan [8]	F, 2	Fever, diarrhoea, vomiting	2	DC (24 h), seizures	136	(+) G1	NE	NE	5	NE	Diazepam PR one dose	NS
											Isotonic fluids	
Natsume et al, 2006 Japan [12]	F, 2	Diarrhoea, vomiting	3	Cluster of seizures	Normal	(+)	NE	NE	9	NAD	Diazepam PR one dose, phenobarbital IM one dose	NS
Fukuda et al, 2009 Japan [10]	M, 2	Fever, diarrhoea, vomiting	3	DC (24 h)	130	(+) G3	NE	(–)	5	GDSW	Midazolam infusion 0.1 mg/kg/h for 1 day	NS
											Methylprednisolone 24 mg/kg/day for 3 days	
Kato et al, 2009 Japan [9]	F, 1	Fever, diarrhoea, vomiting	4	Seizures	137	(+)	NE	(–)	6	Spikes and slow waves in the right occipital area	Diazepam	NS
Jang and Lee, 2010 Korea [16]	F, 2	Fever, diarrhoea, vomiting	2	Seizures	138	(+)	NE	NE	6	NAD	Empiric antibiotics, aciclovir	NS
											Isotonic fluids	
Arakawa et al, 2011 Japan [11]	M, 3	Diarrhoea, vomiting	3	DC (24 h)	128	(+) G9P[8]	(–)	NE	6	NE	Osmotic diuretic, isotonic fluids	NS
Arakawa et al, 2011 Japan [11]	M, 3	Fever, vomiting	3	DC (24 h)	129	(+) G3P[8]	(+) G3	NE	7	NE	Osmotic diuretic, isotonic fluids	NS
Fuchigami et al, 2013 Japan [15]	F, 4	Fever, diarrhoea, vomiting	4	DC (6 days), seizures	140	(+) G1P[8]	(+) G1	(–)	7	GDSW	Osmotic diuretic, isotonic fluids	NS
											Methylprednisolone 30 mg/kg/day for 3 days	
Matsuoka et al, 2013 Japan [17]	M, 4	Diarrhoea, vomiting	4	DC (7 h), seizures	132	(+)/ G5P[6]	(–)	(–)	8	NAD	No treatment	NS
Yokoyama et al, 2013 Japan [13]	M, 2	Fever, diarrhoea, vomiting	2	Seizures	Normal	(+)	NE	(–)	9	NAD	Phenobarbital PO for 9 days	NS
Kashiwagi et al, 2014 Japan [19]	M, 5	Fever, diarrhoea, vomiting	1	DC (32.5 h)	133	(+)	ND	ND	ND	GDSW	Methylprednisolone for 3 days	NS
Mazur-Melewska et al, 2015 Poland [14]	F, 6	Diarrhoea, vomiting	2	Cluster of seizures, DC	137	(+)	NE	NE	12	Slowed waves in the posterior occipito-temporal area	Dexamethasone	NS
											0.4 mg/kg/day for 5 days	
											Isotonic fluids	
Pan et al, 2015 China [18]	F, 2	Fever, diarrhoea, vomiting	ND	Seizures (twice)	134	(+)	ND	ND	5	NAD	Methylprednisolone pulse and oral prednisolone	NS
											Osmotic diuretic, isotonic fluids	
Present case	M, 4	Fever, diarrhoea, vomiting	3	DC (24 h)	131	(+) G1P[8]	NE	(–)	NE	GDSW, more prominent in the right hemisphere	Ceftriaxone, clarithromycin, aciclovir for 5 days	NS
											Isotonic fluids	

*Abbreviations*: *DC* disturbance of consciousness; *GDSW* global diffuse slow waves; *NAD* nothing abnormal detected; *ND* not documented; *NE* not examined; *NS* no sequelae

In the present case, a repeat MRI was not performed, because the SCC lesion was initially thought to represent terminal myelination rather than pathology. The possibility of MERS was considered retrospectively and by this point the patient had fully recovered clinically, such that a repeat MRI scan under general anaesthesia could not be justified.

Other possible differential diagnoses of acquired lesions of the corpus callosum include infarction, tumours (lipoma, lymphoma, glioblastoma), multiple sclerosis, Marchiafava-Bignami disease, trauma-associated diffuse axonal injury, Posterior Reversible Encephalopathy Syndrome (PRES), acute encephalopathy associated with intravenous immunoglobulin therapy, hydrocephalus, antiepileptic drugs (AED), alcoholism, malnutrition, hypoglycemia, hyponatraemia, hypernatraemia and hereditary disorders of myelination (Krabbe's disease, X-linked adrenoleukodystrophy) [20, 21]; however, these were excluded clinically and radiologically in our patient.

Acute disseminated encephalomyelitis (ADEM) should be considered in the differential diagnosis of patients with MERS lesions. In ADEM, the lesions seen in the corpus callosum are usually asymmetrical and contrast-enhancing, extend to the white matter and resolve over weeks to months; by contrast, in MERS the lesions show no contrast enhancement and mostly disappear quickly [4]. Although our patient did not have a repeat brain MRI, the clinical course of his disease makes the diagnosis of ADEM unlikely. An isolated splenial lesion associated with rotavirus gastroenteritis is not always a benign sign. Patients with rotavirus cerebellitis have been reported to have transient splenial changes in the acute phase identical to those found in MERS [22]. Rotavirus cerebellitis concurrent with encephalitis is typically complicated by mutism with subsequent dysarthria and neurological sequelae, and MRI imaging shows chronological changes that involve the cerebellar cortex and result in cerebellar atrophy [22].

Management of rotavirus-associated MERS varies in the literature. Five patients (38 %) were treated with steroids, with no side-effects reported. One patient (8 %) received antimicrobial therapy for suspected meningoencephalitis. Anticonvulsant drugs were given to 5 (38 %) patients mainly as rescue management. Isotonic fluids were given in 7/13 (54 %) cases and an osmotic diuretic in 4/13 (31 %). All patients with rotavirus-associated MERS made a full clinical recovery, so it is difficult to compare the efficacy of these different management approaches. Given its generally benign prognosis, specific immunomodulatory treatment for rotavirus-associated MERS may not be justified. However, supportive care may include antimicrobial cover for more serious causes of meningoencephalitis, such as herpes simplex virus infection, until the diagnosis of MERS is made; control of seizures with anticonvulsants and judicious fluid administration to correct dehydration and electrolyte imbalances secondary to rotavirus gastroenteritis, particularly since hyponatraemia has been hypothesized to contribute to the pathogenesis of MERS.

The pathogenesis of rotavirus-associated neurological disease is still incompletely understood. Detection of rotavirus RNA by PCR from CSF of some patients with neurological complications supports a hypothesis of direct viral invasion [23]. However, the significance of this finding remains unclear. It has generally been believed that rotavirus is localized to the epithelial cells of the small intestine, although a series of studies has demonstrated that rotavirus infection in children results in antigenaemia and viraemia during the acute phase of the disease [2]. These findings highlight the fact that extra-intestinal dissemination of rotavirus can occur, but the association between rotavirus viraemia and CNS complications is not clearly demonstrated. Detection of rotavirus RNA in CSF did not correlate with the presence of rotavirus antigen in CSF in a study by Nakagomi and Nakagomi [24]. It is also possible that CSF contamination with diarrhoeal stools could occur at the time of lumbar puncture or during laboratory handling [23]. There are no reports of detection of rotavirus RNA from the CSF of patients with rotavirus-associated MERS, suggesting that direct invasion is unlikely to be the pathogenic mechanism. In addition, MERS is also caused by other pathogens, including influenza virus A and B, mumps virus and adenovirus [6].

It has been hypothesised that intramyelinic oedema or inflammation of the corpus callosum may play a role in the pathogenesis of MERS [7]. Since hyponatraemia is common in MERS, including the present case, cerebral oedema secondary to hypotonic dehydration is a possible mechanism. This hypothesis is supported by data from two other conditions. First, a completely reversible splenial lesion has been described in patients on antiepileptic drugs, usually when the dose is reduced quickly, possibly caused by a transient change in water balance due to the effect these drugs have on electrolyte channels [25]. Second, similar splenial changes have been found in patients with high-altitude cerebral oedema, where hypoxia causes vasogenic interstitial oedema [26]. A water-electrolyte imbalance may therefore be a common mechanism underlying these three conditions; however, hyponatraemia is not universal in cases of MERS [7]. Another possible explanation is a local infiltration of inflammatory cells. Interleukin-6 (IL-6) and nitric oxide metabolites may have an important role in the pathophysiology of MERS [27]. Although elevated serum IL-6 appears to have good predictive value for the clinical severity of influenza virus-associated encephalopathy [28], IL-6 measurements have not been performed in sufficient MERS cases for any conclusions to be drawn. A small study showed increased CSF levels of IL-6 and IL-10 in three of six patients with MERS, of whom two had lesions extending to the white matter [27]. The rotavirus non-structural protein 4 (NSP4) induces nitric oxide metabolites, which are highly reactive free radicals [29]. Nitrites and nitrates have been reported to be raised in both serum and CSF of patients with rotavirus-associated seizures, but the report in this study did not describe outcomes based on the presence or absence of splenial changes [30]. Taken together, MERS is a rare syndrome of uncertain pathogenesis; despite a number of plausible hypotheses, none explains why the splenium is the site that is specifically involved.

Rotavirus immunisation is now recommended by the World Health Organization, and has recently been introduced in the UK. Payne and colleagues [31] reported an unexpected benefit of rotavirus vaccination in the USA. A retrospective analysis of 250,601 infants showed that a full rotavirus vaccination course was associated with an 18–21 % decrease in the risk of seizures during the first year after vaccination, suggesting that rotavirus infection may be an under-recognized cause of neurological disease. Extrapolating findings from this study, it is biologically plausible that rotavirus vaccination may also reduce other neurological manifestations of rotavirus infection. With the exception of one case, which was associated with the G5P6 genotype, all other reported cases of rotavirus-associated MERS (including the present one) were associated with rotavirus genotypes that are covered by the available vaccines.

## Conclusions

Although the burden of disease has decreased significantly in countries where vaccination has been introduced, rotavirus is still a common cause of hospitalization [32] in developed countries and it should be considered in the differential diagnosis of a child with encephalopathy or encephalitis, particularly if associated with a diarrhoeal disease [33]. MERS has been increasingly recognized as a clinical entity in Japan, where it was first described, with only few cases reported to date outside of East Asia. A recent study by Ka *et a*l reported seven cases of MERS in Caucasian Australian children, related to various infectious or inflammatory triggers, although none had rotavirus infection [34]. It is likely that MERS is under-diagnosed, since the diagnosis of this condition relies on MRI. The outcome of rotavirus-associated MERS is good, with the majority of patients achieving full recovery irrespective of treatment. However, it is important to be aware that rotavirus infection is also associated with more severe neurological complications, such as acute cerebellitis, which might also present with an isolated splenial lesion. This case, together with the other published case reports, supports the hypothesis that rotavirus-associated MERS is unlikely to be the result of direct viral invasion of the CNS. However, the pathogenesis of MERS remains unclear, and a further understanding of its mechanisms is required.

## Consent

Written informed consent was obtained from the parents of our patient for publication of this case report and any accompanying images. A copy of the written consent is available for review by the Editor of this journal.

## Abbreviations

MERS: Mild Encephalitis/Encephalopathy with a reversible splenial lesion
MRI: Magnetic resonance imaging
CT: Computed tomography
CNS: Central nervous system
CSF: Cerebrospinal fluid
SCC: Splenium of the corpus callosum
ADC: Apparent diffusion coefficient
EEG: Electroencephalography
LP: Lumbar puncture
PCR: Polymerase chain reaction
ADEM: Acute disseminated encephalomyelitis
AEDs: Antiepileptic drugs
NSP4: non-structural protein 4
DC: Disturbance of consciousness
GDSW: Global diffuse slow waves
NAD: Nothing abnormal detected
ND: Not documented
NE: Not examined
NS: No sequelae
